# ATR-FTIR spectrum analysis of saliva samples from COVID-19 positive patients

**DOI:** 10.1038/s41598-021-99529-w

**Published:** 2021-10-07

**Authors:** Adriana Martinez-Cuazitl, Gustavo J. Vazquez-Zapien, Miguel Sanchez-Brito, Jorge H. Limon-Pacheco, Melissa Guerrero-Ruiz, Francisco Garibay-Gonzalez, Raul J. Delgado-Macuil, Maria G. Garcia de Jesus, Miguel A. Corona-Perezgrovas, Armando Pereyra-Talamantes, Monica M. Mata-Miranda

**Affiliations:** 1 Escuela Militar de Medicina, Centro Militar de Ciencias de la Salud, Secretaría de la Defensa Nacional, 11200 Mexico City, Mexico; 2 Hospital Central Militar, Secretaría de la Defensa Nacional, 11200 Mexico City, Mexico; 3 TecNM/Instituto Tecnológico de Aguascalientes, 20256 Aguascalientes, Mexico; 4 CIBA-Tlaxcala, Instituto Politécnico Nacional, 90700 Tlaxcala, Mexico; 5 Universidad Politécnica de Cuautitlán Izcalli, 54760 Estado de México, Mexico

**Keywords:** Medical research, Biologics

## Abstract

The coronavirus disease 2019 (COVID-19) is the latest biological hazard for the novel severe acute respiratory syndrome coronavirus 2 (SARS-CoV-2). Even though numerous diagnostic tests for SARS-CoV-2 have been proposed, new diagnosis strategies are being developed, looking for less expensive methods to be used as screening. This study aimed to establish salivary vibrational modes analyzed by attenuated total reflection-Fourier transform infrared (ATR-FTIR) spectroscopy to detect COVID-19 biological fingerprints that allow the discrimination between COVID-19 and healthy patients. Clinical dates, laboratories, and saliva samples of COVID-19 patients (N = 255) and healthy persons (N = 1209) were obtained and analyzed through ATR-FTIR spectroscopy. Then, a multivariate linear regression model (MLRM) was developed. The COVID-19 patients showed low SaO_2_, cough, dyspnea, headache, and fever principally. C-reactive protein, lactate dehydrogenase, fibrinogen, d-dimer, and ferritin were the most important altered laboratory blood tests, which were increased. In addition, changes in amide I and immunoglobulin regions were evidenced in the FTIR spectra analysis, and the MLRM showed clear discrimination between both groups. Specific salivary vibrational modes employing ATR-FTIR spectroscopy were established; moreover, the COVID-19 biological fingerprint in saliva was characterized, allowing the COVID-19 detection using an MLRM, which could be helpful for the development of new diagnostic devices.

## Introduction

The coronavirus disease 2019 (COVID-19) is the latest biological hazard provoked for the novel severe acute respiratory syndrome coronavirus 2 (SARS-CoV-2), which is wreaking havoc in the health, political, economic, and social sectors around the world^1^. More than 180 countries are affected by this pandemic, and despite all containment efforts, the number of COVID-19 infected people is rising above 160 million, with over three million deaths^2,3^. The mean of incubation period and serial intervals are 5.2 and 7.5 days, respectively, while the basic reproduction number (R_0_) is 2.2 persons on average^4^.

Coronaviruses are single-stranded enveloped RNA viruses that cause diseases in mammals and birds. They are composed of the nucleocapsid (N) and spike (S) proteins, which participate in viral genome assembly, transcription, replication, or mediating viral entry provoking a cytopathic effect^5^.

Some diagnostic tests for SARS-CoV-2 infection, such as the use of nucleic acids amplification test, antigen test, and serological antibody immunoassays, have been proposed and used. However, even though the reverse transcriptase-polymerase chain reaction (RT-PCR) is considered the gold standard for detecting SARS-CoV-2, it can produce false-negative results. Moreover, the RT-PCR testing procedure requires high-level personnel training and a high level of laboratory expertise. It usually takes 4–6 h to complete the test, highlighting that this technique is expensive, making it unsuitable for screening^3,6^.

On the other hand, antigen-based diagnostic tests employing monoclonal antibodies against SARS-CoV-2 antigens, such as N protein and the S1 or S2 domains of the S protein, can also be used to detect viral infection. Nevertheless, these are less sensitive than RT-PCR tests, but an advantage is that they are available in rapid test kits, providing results in 20–60 min, and are easy to use. Enthought, it is a low-cost alternative that can be widely used at the community level; it may take 21 days or longer after symptom onset for seroconversion or detection of antibodies to SARS-CoV-2, like immunoglobulin (Ig) M and/or IgG, which have shown 88.66% of sensitivity and 90.63% of specificity in blood samples^3,6^.

New diagnosis strategies are being developed, looking for less expensive methods that could be used as screening for better spread control and greater sensitivity and specificity. Moreover, even though nasopharyngeal testing specimens remain the current standard for SARS-CoV-2 diagnosing, lower respiratory tract samples have a higher yield than upper tract samples, but they are often not obtained due to aerosolization of the virus during sample collection procedures. For this reason, some other tests suggest the use of saliva specimens^3^.

Considering the above mentioned, in this research, we propose the Fourier transform infrared (FTIR) spectroscopy technique, which analyzes the components of saliva samples emitting a "biological fingerprint", allowing the discrimination between positive and negative COVID-19 patients. FTIR is a technique based on the measurement of the absorption of electromagnetic radiation with wavelengths within the mid-infrared (IR) region (4000–400 cm^−1^) that identifies variations in functional groups through the measure of the vibration and rotation of molecules influenced by IR^7^, providing information of the structure and chemical composition (lipids, proteins, carbohydrates, and nucleic acids) concerning biological samples, like tissues, cells, and biological fluids^8^.

The reason for using saliva is the easy way of sampling, once the same patient can recollect it, without the complications (bruising, mucosal erosions, and bleeding) showed in nasopharyngeal and oropharyngeal swabs. Besides, saliva shows viral shedding of both the salivary glands and the upper and lower respiratory tract. Moreover, previous studies have demonstrated that saliva has a high concordance rate with more than 90% with nasopharyngeal samples in detecting respiratory viruses, including coronaviruses^9^. In this sense, the use of saliva as a potential indicator of COVID-19 has been described by many researchers^10–15^.

However, the people characterization from the FTIR spectra analysis is complex due to the spectra differences, even from the same group. Moreover, saliva has a significant content of components, which show an intersection of its links, making it challenging to describe particular affectations of pathologies^16^. Currently, the problem of characterizing populations from FTIR spectra has been addressed using techniques commonly employed in the area of machine learning; Santos et al. have suggested the practice of different methodologies that allow distinguishing between two or more groups of spectra based on the results obtained through them in different investigations^17^. However, since there is no way to know which method would be the most appropriate to tackle a particular problem, some authors have suggested using regression models followed by more complex models such as vector support machines (SVM) or artificial neural networks (ANN).

The present study aimed to establish specific salivary vibrational modes analyzed by attenuated total reflection-Fourier transform infrared (ATR-FTIR) spectroscopy to detect COVID-19 biological fingerprints suitable for diagnosis through a multivariate linear regression model (MLRM), allowing the discrimination between COVID-19 and healthy patients.

## Materials and methods

### Ethical aspects and study subjects

In this study, we discriminated between positive and negative COVID-19 patients through ATR-FTIR spectroscopy. For that purpose, the healthy group samples were used as a control, and it was integrated by 1209 healthy ambulatory volunteers who were recruited from February 2019 to February 2020 (a period in which the COVID-19 has not reached Mexico borders), 496 (41%) men, and 713 (59%) women with an average age of 60.5 ± 8.6 years. For positive COVID-19 samples, 255 hospitalized patients positive to SARS-CoV-2 diagnosticated through RT-PCR technique were recruited from May 2020 to March 2021, 160 (62.7%) men, and 95 (37.2%) women with an average age of 54.3 ± 14.7 years. Written informed consent for the obtention of 1 mL of saliva and participation in this study was obtained from the participants. The diagnosis of COVID-19 was developed in the Hospital Central Militar, Mexico.

The inclusion criteria were patients that accepted to participate in this study, aged over 18 years, and fasting at least 8 h. The exclusion criteria were patients who brushed or rinsed the oral cavity with mouthwash before sampling and patients with orthodontic or other dental treatments. The healthy patients were informed that their samples would be used for different diagnostic assays as a reference control, and the COVID-19 patients were informed that their samples would be used to try other types of diagnosis.

The Clinical Research Ethics Committee of the Unidad de Especialidades Medicas and the Hospital Central Militar of the Secretaria de la Defensa Nacional approved the protocol and informed consent.

### COVID-19 patients clinical data compilation

The 72.2% of the COVID-19 population were sampled in the first three weeks of hospitalization; besides, the samples were collected on day 9.24 after RT-PCR diagnosis, highlighting that only one sample of each patient was obtained for the development of this research which was analyzed immediately without storage need. At the sampling moment, vital signs were evaluated, symptoms like cough, dyspnoea, headache, fever, myalgia, arthralgia, among others, were interrogated, as well as comorbidities such as diabetes, obesity, hypertension, smoking, and other of importance in this disease. In the same way, the blood type was questioned. Finally, laboratory blood tests (hematic biometry, blood chemistry test, serum electrolytes, hepatic-function test, blood gas test, and others) were evaluated.

### Sample pre-processing

For FTIR spectral analysis, we develop the methodology that has been carried out for the analysis of biological samples^18^. Therefore, this pre-processing was conducted in the spectral range between 4000 and 400 cm^−1^ (mid-infrared), using an FTIR spectrometer (6600, Jasco) in the attenuated total reflection (ATR) sampling mode. The instrument has a fixed spectral resolution of 4 cm^−1^. Three µL of each sample was deposited onto the surface of the ATR crystal and dried at room temperature for about 15 min to eliminate excess water. The IR radiation propagated along the crystal to obtain the corresponding spectra that were the average of 120 data acquisitions. Each sample was analyzed three times, and all data were obtained in triplicate.

### Spectral analysis

After normalizing through standard normal variate (SNV) and calculating the spectra' second derivative, the analysis was performed in the biological fingerprint (1800–800 cm^−1^). The second derivative was obtained using Unscrambler X. Next, the mean of each population was obtained to identify relevant differences (absorbance differences and displacements). The graphs were obtained employing the Origin software (version 6.0, OriginLab Corporation).

### Immune response and DNA and nucleic acids content through FTIR

Like other pathogens that cause infections, SARS-CoV-2 infection causes IgM, IgG, and IgA antibodies, making it mandatory to evaluate and compare the concentration of these antibodies between the COVID-19 group and the healthy group. For which purpose, the integrated areas were assessed at 1420–1289 cm^−1^ and 1160–1028 cm^−1^ regions to evaluate IgM, 1560–1464 cm^−1^ which correspond to IgG, and finally, the area at 1285–1237 cm^−1^ corresponded to IgA^19^. Once the integrated areas were calculated, a Mann–Whitney test was developed to determine any significant differences between COVID-19 and the healthy group.

Moreover, the integrated areas of IgA, IgM, and IgG were compared between them in the COVID-19 group, employing a Kruskal–Wallis test.

The DNA and nucleic acid content were also compared between COVID-19 and healthy groups, analyzing the ratio (A968/992) for DNA content and the integrated area at 1237 cm^−1^ for nucleic acid content^20^. After that, a T-student test was developed to determine any significant differences.

### Classification model

For the classification of the population, we carry out a multivariate linear regression model (MLRM), which is similar to the simple regression model except for having more coefficients for the variables considered (1) where *b* is the interceptor, m is the slope, and *x* an absorbance value:

1 $$ {\text{Y}}_{{\text{i}}} = {\text{ b }} + {\text{ m}}_{{1}} {\text{x}}_{{1}} + \, \cdots \, + {\text{ m}}_{{\text{n}}} {\text{x}}_{{\text{n}}}  $$

Regression analysis is a statistical technique for investigating and modeling the relationship between variables. This analysis is called a multivariate or multiple linear regression model because more than one regressor is involved.

We used the leave-one-out (LOOCV) cross-validation methodology to evaluate the classification model because it has less bias than a validation set and produces the same results each time you run it^21^. The LOOCV methodology considers segmenting the database into two subsets: training and evaluation. The training subset comprises N − 1 samples, and the evaluation subset considers the omitted spectrum in the training process. These partitions are performed as many times as spectra make up the database; in this way, all spectra contribute N − 1 times to the regression model, and all spectra are evaluated once.

### Ethics approval and consent to participate

Written informed consent for the obtention of 1 mL of saliva and participation in this study was obtained from the participants. The Clinical Research Ethics Committee of the Unidad de Especialidades Medicas and the Hospital Central Militar of the Secretaria de la Defensa Nacional approved the protocol and informed consent. Furthermore, all experiments were examined and approved by the appropriate ethics committee; besides, ethical standards of the 1964 Declaration of Helsinki were followed.

### Consent for publication

The authors give consent for publication.

## Results

### Study population description

As previously mentioned, the healthy group was integrated by 1209 patients, 496 (41%) men, and 713 (59%) women with an average age of 60.5 ± 8.6 years. Furthermore, the COVID-19 group was integrated by 255 patients, 160 (62.7%) men and 95 (37.3%) women with an average age of 54.3 ± 14.7 years. Focusing on the COVID-19 group, the signs and symptoms, comorbidities, blood type, and laboratory blood tests were evaluated.

Table 1 shows the signs and symptoms that each patient presented and referred. The average of the obtained vital signs showed that the patients presented a low SaO_2_ (oxygen saturation); however, the rest of the vital signs were within normal parameters. Likewise, the ponderal state was determined through body mass index (BMI), evidencing overweight. The main symptoms that the patients mentioned were cough, dyspnoea, headache, and fever in more than 50% of the patients, followed by myalgias and arthralgias in more than 45% of the patients.

**Table 1:** Signs and symptoms.

Vital signs and ponderal state	Median (IQR)
SaO_2_ (%)	90 (83, 94)
Heart rate (beats per minute)	87 (77, 102)
Respiratory rate (breaths per minute)	22 (20, 25)
Systolic (mmHg)	120 (108, 131)
Diastolic (mmHg)	72 (65, 80)
Mean arterial pressure (mmHg)	94 (86, 103)
BMI (kg/m^2^)	28.3 (25.5, 35.1)

Symptoms	n (%)
Cough	194 (76.1)
Dyspnoea	189 (74.1)
Headache	145 (56.9)
Fever	145 (56.9)
Myalgia	125 (49)
Arthralgia	120 (47.1)
Sputum	67 (26.3)
Pharyngeal pain	80 (31.4)
Emesis	30 (11.8)
Conjunctivitis	31 (12.2)
Asymptomatic	11 (4.3)

Table 2 resumes the comorbidities showed in the COVID-19 group, where it can be observed that 186 patients (72.9%) presented comorbidities, and some of them showed more than two comorbidities; even more, 2.4% of the patients presented more than four comorbidities. Obesity was the main comorbidity, the 38% of the COVID-19 patients showed this condition followed by diabetes and hypertension. Table 3 shows that the main blood type of the population that integrated the COVID-19 group was O+ (61.6%).

**Table 2:** Comorbidities.

Comorbidities	n (%)
Obesity	97 (38)
Diabetes	84 (32.9)
Hypertension	70 (27.5)
Smoking	45 (17.6)
HIV	3 (1.2)
Cardiovascular disease	4 (1.6)
Others (cancer, Parkinson, infertility)	3 (1.2)
Asthma	2 (0.8)
Tuberculosis	2 (1.2)
None	69 (27.1)

**Table 3:** Blood type.

Blood type	n (%)
O+	157 (61.6)
O−	2 (0.8)
A+	32 (12.5)
A−	2 (0.8)
B+	7 (2.7)
Unknown	55 (21.6)

The laboratory blood tests findings are described in Table 4, where it can be observed that the median values of neutrophils, N/L ratio, glucose, blood pH, C-reactive protein (CRP), lactate dehydrogenase (LDH), fibrinogen, d-dimer, and ferritin were increased; contrary PaO_2_ and PaCO_2_ were diminished.

**Table 4:** Laboratory blood tests.

	n	Median (IQR)
**Hematic biometry**		
Leukocytes (10^3^/µL)	252	8.4 (6.3, 11.8)
Neutrophils (10^3^/µL)	238	9.9 (5.6, 6.7)
Lymphocytes (10^3^/µL)	249	1.2 (0.75, 3.7)
N/L ratio	243	8.1 (4.4, 14.7)
Hemoglobin (g/dL)	251	15.3 (14, 16.4)
Platelets (10^3^/µL)	251	238 (168.8, 318.5)
**Blood chemistry test**		
Creatinine (mg/dL)	250	0.8 (0.6, 0.96)
Urea (mg/dL)	240	34.6 (27.0, 49.2)
Glucose (mg/dL)	241	126 (103.3, 189.8)
**Serum electrolytes**		
Na (mmol/L)	240	139 (137, 141)
K (mmol/L)	240	4.4 (4, 5)
Mg (mmol/L)	174	2.1 (2.0, 2.4)
**Hepatic-function test**		
ALT (U/L)	244	38 (25, 64)
AST (U/L)	243	39 (29, 55)
ALP (U/L)	146	91 (69, 124)
Bilirubin, total (mg/dL)	189	0.8 (0.6, 1.1)
Albumin (g/dL)	239	3.3 (3, 3.7)
**Blood gas test**		
pH	150	7.46 (7.43, 7.50)
PaO_2_ (mmHg)	149	66 (54, 77)
PaCO_2_ (mmHg)	148	31.1 (28, 36)
HCO^3−^ (mmol/L)	149	22.7 (20.6, 24.7)
Lactate (mmol/L)	149	1.4 (0.9, 1.9)
**Other**		
CRP (mg/dL)	228	90 (43.2, 202.4)
LDH (UI/L)	144	397 (266.3, 595.8)
Fibrinogen (mg/dL)	121	693 (467.3, 825.8)
d-dimer (ng/mL)	140	777 (438, 1675)
Ferritin (ng/mL)	143	489.5 (287.8, 954)

### FTIR analysis

**Figure 1:**
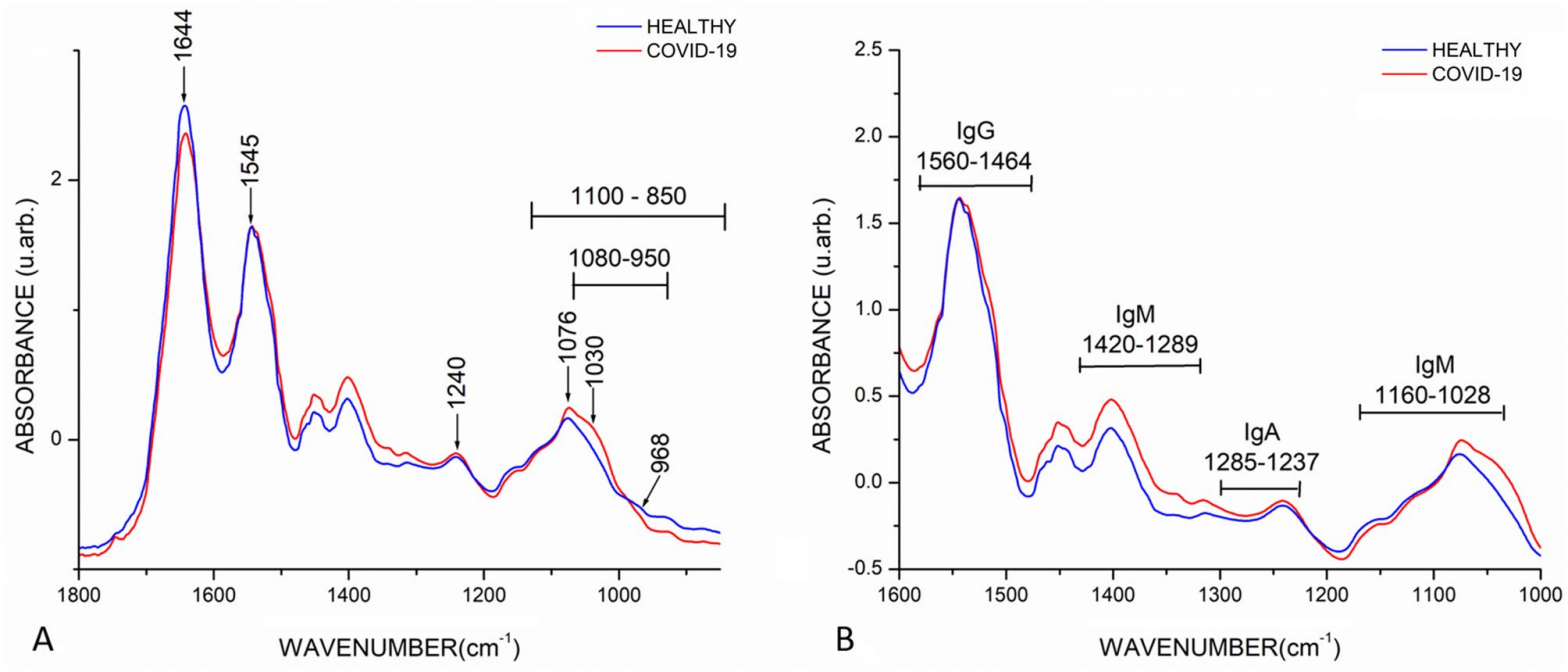
Mean of FTIR spectra of healthy (N = 1209) and COVID-19 (N = 255) groups. (**A**) Biological fingerprint region, diverse absorption bands related to biological samples are evidenced such as Amide I (1644 cm^−1^), Amide II (1545 cm^−1^), and Amide III (1240 cm^−1^), as well as phosphorylated molecules (1240 cm^−1^ and 1076 cm^−1^), carbohydrates (1030 cm^−1^), and DNA backbone (968 cm^−1^). Likewise, ranges at 1100–850 cm^−1^ and 1080–950 cm^−1^ attributed to nucleic acids and α-amylase respectively were observed. Differences in absorbance and displacements between the bands of the groups representing changes in biochemical compositions were evidenced. (**B**) Immunoglobulins regions, different immunoglobins intervals were detected such as IgG (560–1464 cm^−1^), IgM (1420–1289 cm^−1^ and 1160–1028 cm^−1^), and IgA (1285–1237 cm^−1^), noticing that the COVID-19 group exhibited a higher absorbance.

Figure 1A shows saliva FTIR spectra of COVID-19 (red spectrum) and healthy (blue spectrum) groups, where diverse absorption bands related to different biomolecules are evidenced, such as lipids, proteins, carbohydrates, and nucleic acids, usually present in biological samples. The average saliva spectrum of both groups showed characteristics of biological samples, peaks of proteins at 1644 cm^−1^ (Amide I, C=O stretching), 1545 cm^−1^ (Amide II, N–H bending), and 1240 cm^−1^ (Amide III) were evidenced. Besides, in the region of nucleic acids (1100–850 cm^−1^), P=O asymmetrical and symmetrical stretching vibrations of PO_2_ phosphodiester groups from phosphorylated molecules (1240 cm^−1^ and 1076 cm^−1^) were also observed, as well as C–O stretching vibration coupled with C–O bending of the C–OH groups of carbohydrates (including glucose, fructose, and glycogen) at 1030 cm^−1^. Moreover, a band at 968 cm^−1^ from the DNA backbone stretching vibration is also demonstrated. Finally, the sugar moieties of glycosylated proteins, including α-amylase, were shown in the spectral range of 1080–950 cm^−1^. In this figure, it was possible to distinguish differences in absorbance and displacements between the bands of the groups, representing changes in biochemical compositions mainly in the bands related to phosphorylated molecules and carbohydrates.

Moreover, in the biological fingerprint region, the following intervals related to immunoglobins were detected, 1560–1464 cm^−1^ associated to IgG, 1420–1289 cm^−1^ and 1160–1028 cm^−1^ related to IgM, and 1285–1237 cm^−1^ designed to IgA (Fig. 1B), evidencing that the COVID-19 group exhibited higher absorbances than the healthy group.

**Figure 2:**
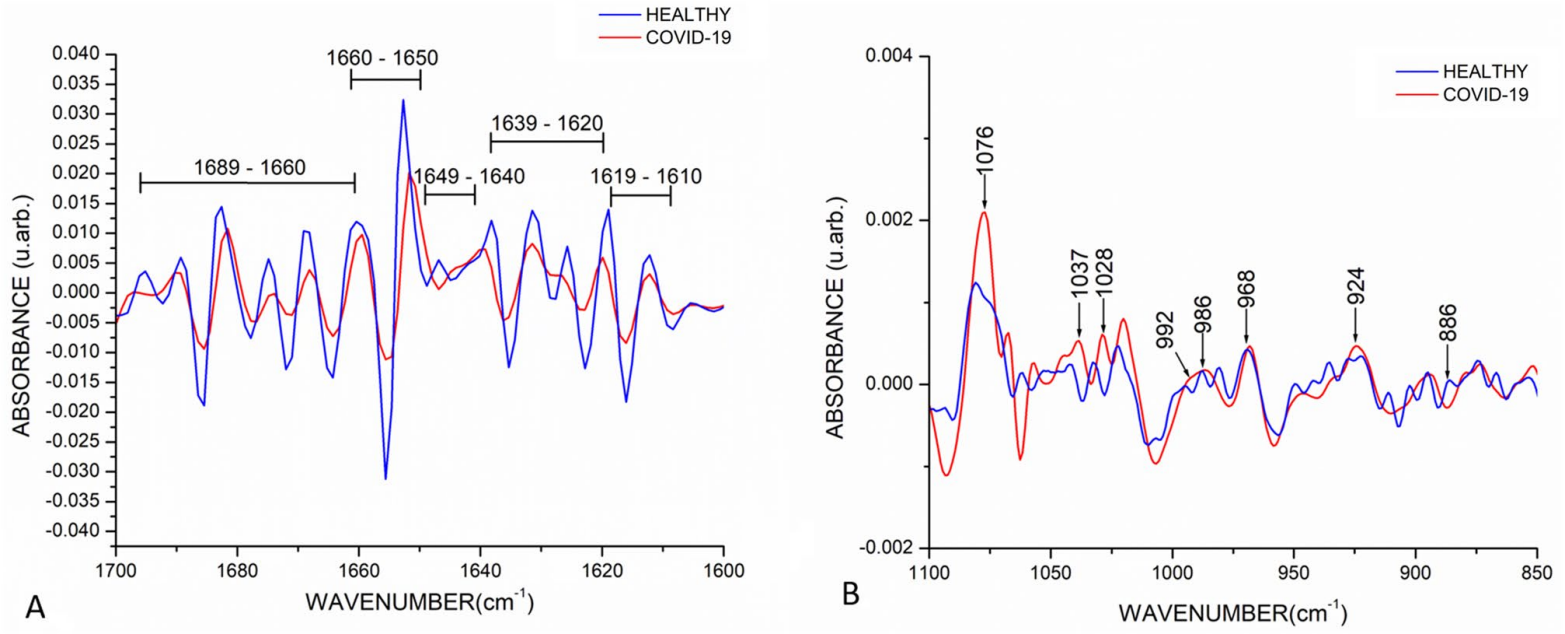
Mean of FTIR second derivative spectra of healthy (N = 1209) and COVID-19 (N = 255) groups. (**A**) Amide I region (1700–1600 cm^−1^), bands related to the components of the secondary structure of proteins are shown, such as β-turns (1695–1660 cm^−1^), α-helices (1660–1650 cm^−1^), intramolecular native β-sheets (1640–1630 cm^−1^), and intermolecular β-sheets (1625–1615); herein an increment in the intensity of these bands in the healthy group compared with the COVID-19 group is observed. (**B**) Nucleic acids region (1100–850 cm^−1^), bands corresponding to symmetrical stretching vibrations of PO_2_ phosphodiester groups (1076 cm^−1^), glycogen (1037 cm^−1^ and 1028 cm^−1^), stretching vibration C–C of DNA backbone (968 cm^−1^), and vibration of ribose ring (924 cm^−1^) showed an increment in the intensity in the COVID-19 group. Moreover, the bands related to the ribose phosphate main chain (992 cm^−1^) and stretching vibration C–C of DNA backbone (986 cm^−1^) exhibited a change in their conformation in the COVID-19 group.

Furthermore, to analyze biomolecular changes with greater precision, we compared the spectra in the second derivative. Figure 2A shows the second derivative of the FTIR spectra of the COVID-19 and healthy groups depicted in the amide I region (1700–1600 cm^−1^), where the bands related to the components of the secondary structure of proteins are shown, such as β-turns (1689–1660 cm^−1^), α-helices (1660–1650 cm^−1^), random coil structure (1649–1640 cm^−1^), β-sheets (1639–1620 cm^−1^), and intermolecular β-sheets (1619–1610) which are sensitive to structural and conformational changes^22^. Notably, the COVID-19 group showed a lower absorbance than the healthy group.

In the same way, Fig. 2B shows the second derivative of the region of the nucleic acids (1100–850 cm^−1^), where a significant increasement in the intensity of the band corresponding to symmetrical stretching vibrations of PO_2_ phosphodiester groups at 1076 cm^-1^ is evidenced; similarly, the bands at 1037 cm^-1^ and 1028 cm^−1^ associated to glycogen showed a higher absorbance in the COVID-19 group. On the other hand, the bands at 992 cm^−1^ and 986 cm^−1^ associated ribose phosphate main chain and stretching vibration C–C of DNA backbone, respectively, changed their conformation in the COVID-19 group. Finally, the bands at 968 cm^−1^ and 924 cm^−1^ associated with the stretching vibration C–C of DNA backbone and the vibration of ribose ring increased in the COVID-19 group.

### Comparison of immunoglobulins, DNA, and nucleic acids content

**Figure 3:**
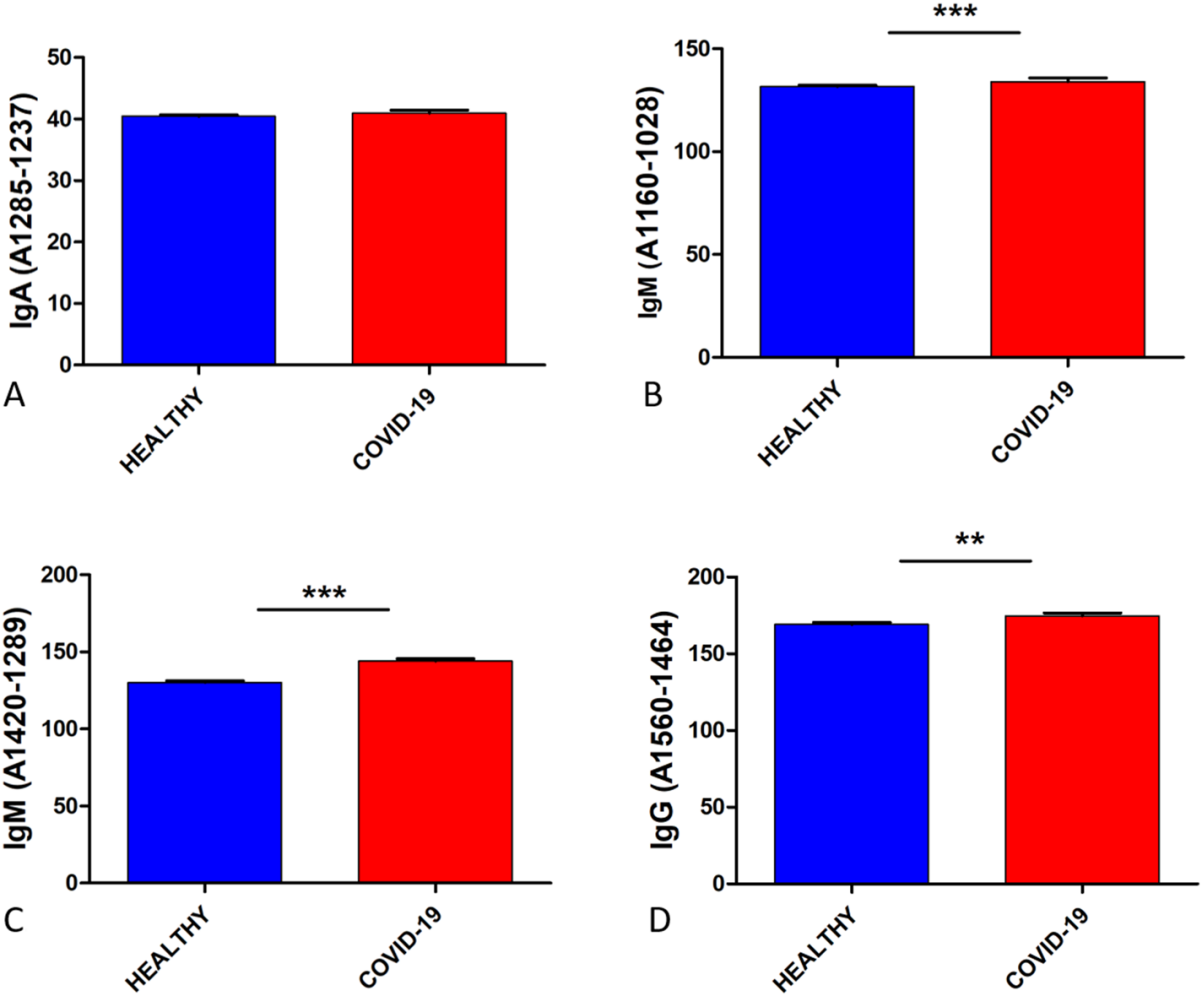
Immunoglobins content comparison between healthy (N = 1209) and COVID-19 (N = 255) groups. (**A**) IgA (1285–1237 cm^−1^), (**B**) IgM (1160–1028 cm^−1^), (**C**) IgM (1420–1289 cm^−1^), (**D**) IgG (1560–1464 cm^−1^). The three immunoglobulins showed a higher expression in the COVID-19 group, showing statistical significance IgM and IgG. Means of immunoglobins expression with error bars representing the standard deviation are depicted, **p < 0.05 and ***p < 0.005, compared to the healthy group.

Figure 3 shows the content of IgA (1285–1237 cm^−1^), IgM (1420–1289 cm^−1^, 1160–1028 cm^−1^), and IgG (1560–1464 cm^−1^) in healthy and COVID-19 groups. The IgA was slightly higher in the COVID-19 group (40.95) than the healthy group (40.42), not showing statistical significance. Likewise, IgM (134.10 and 144.03) in the two studied regions and IgG (174.80) were more expressed in the COVID-19 group compared to the healthy group, IgM (131.69 and 130.24) and IgG (169.14), showing statistical significance.

**Figure 4:**
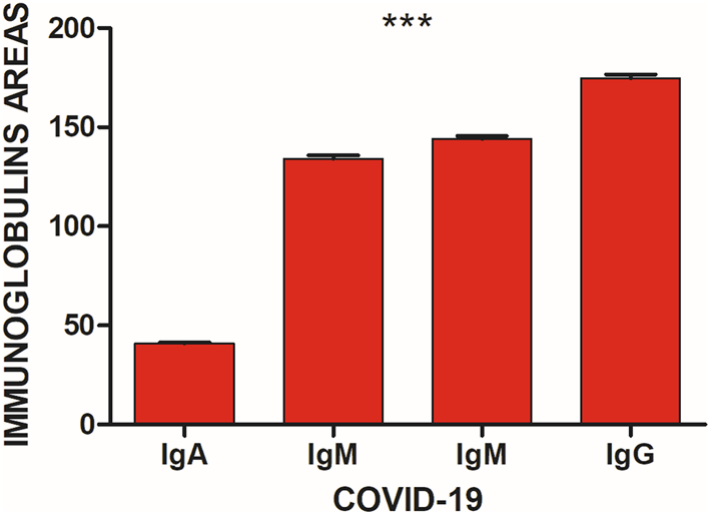
Immunoglobins expression in COVID-19 group (N = 255). IgA (1285–1237 cm^−1^), IgM (1160–1028 cm^−1^, 1420–1289 cm^−1^), IgG (1560–1464 cm^−1^). The IgA was the lowest expressed immunoglobulin, and the most expressed was de IgG, showing a statistical significance between each immunoglobulin. Means of immunoglobins expression with error bars representing the standard deviation are depicted, ***p < 0.005, compared between each immunoglobin.

When comparing the expression of the immunoglobins in the COVID-19 group, it can be observed that the IgA was the lowest expressed immunoglobulin, and the most expressed in this research group was de IgG, showing a statistical significance between each immunoglobulin (Fig. 4).

**Figure 5:**
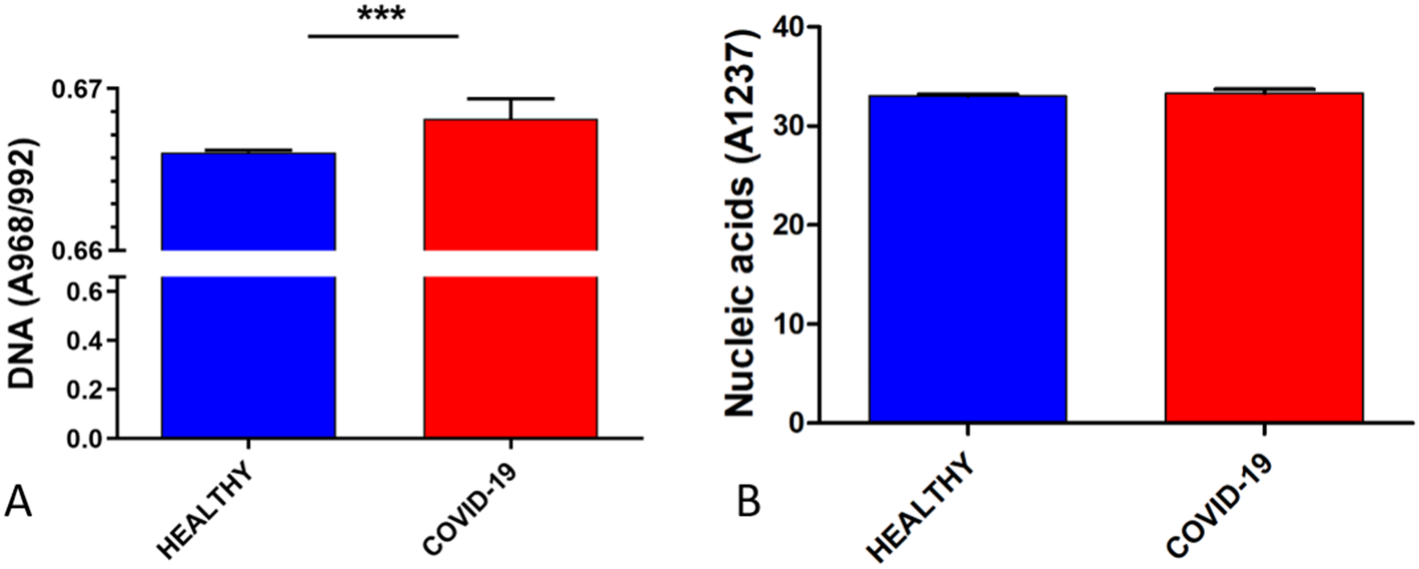
Comparison of the DNA and nucleic acids content between healthy and COVID-19 groups. (**A**) DNA content, the COVID-19 group exhibited a higher DNA content (0.6681) compared to the healthy group (0.6660). (**B**) Nucleic acids content, the COVID-19 group showed a higher nucleic acids content (33.30) relative to the healthy group (33.00). Means of DNA and nucleic acids content with error bars representing the standard deviation are depicted, ***p < 0.005.

Figure 5 depicts DNA and nucleic acids content, where it can be observed that the DNA content exhibited a higher expression in the COVID-19 group (0.6681) than the healthy group (0.6660), showing statistical significance. In the same way, the content of the nucleic acid exhibited a higher expression in the COVID-19 group (33.30) compared to the healthy group (33.00); nevertheless, no statistical significance was observed.

### Multivariate analysis

**Figure 6:**
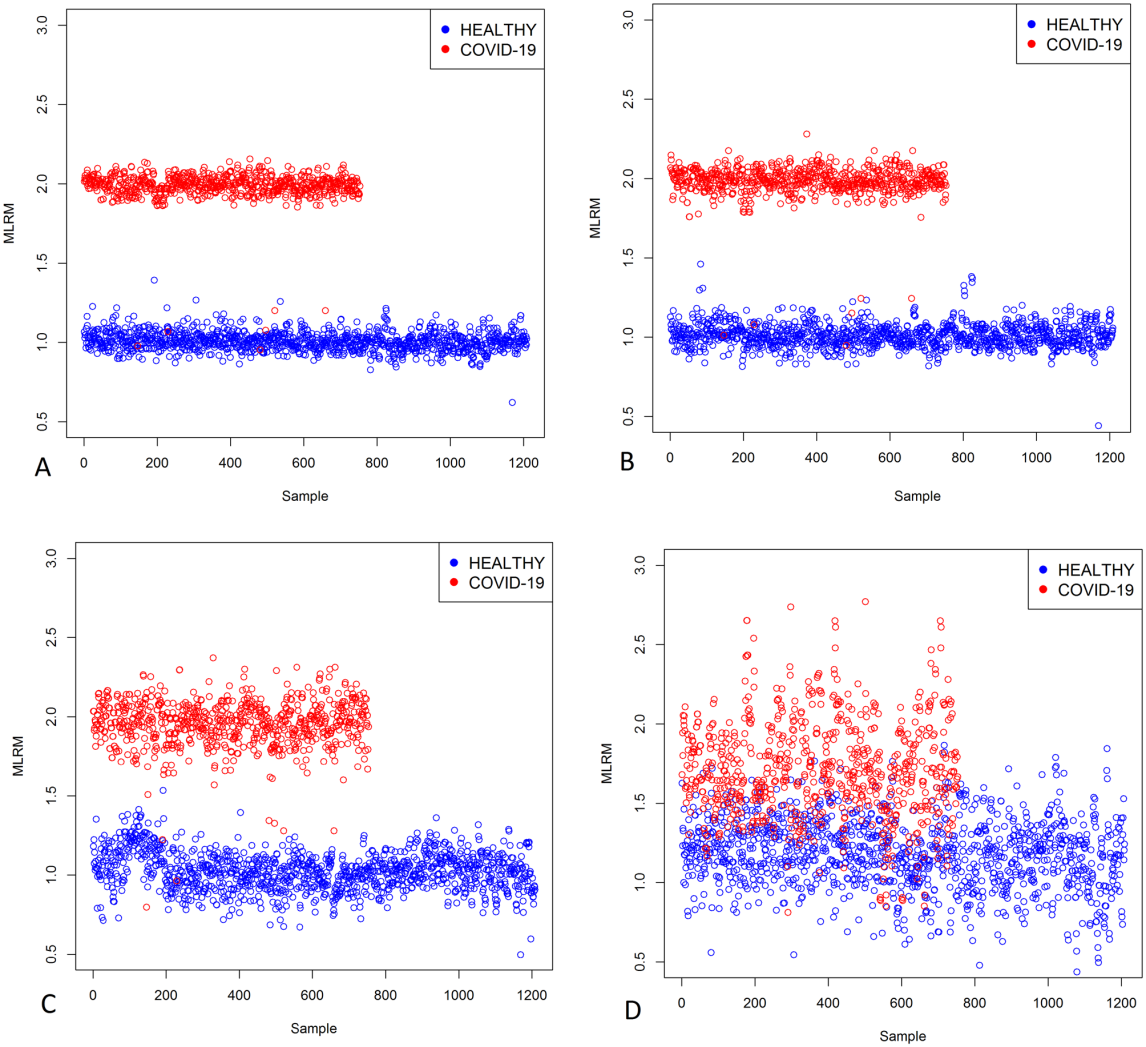
Multiple linear regression model (MLRM) between healthy (N = 1209) and COVID-19 (N = 255) groups in the regions of Amide I, IgG, and IgM. (**A**) Amide I (1700–1600 cm^−1^), the outputs data are very compact for each population, and the distance between them is well defined, allowing their discrimination. Even though some data from the COVID-19 group are badly grouped and located in the healthy group, the MLRM in the amide I was the best region to discriminate COVID-19 from healthy persons. (**B**) IgG (1560–1464 cm^−1^), the depicted data are grouped in two populations, allowing the discrimination between healthy and COVID-19 groups; nevertheless, some data of both populations are ungrouped, locating COVID-19 data in the healthy group. (**C**) IgM (1420–1289 cm^−1^), the outputs are clustered, but the distance between the groups is not well defined, not allowing the identification of groups. (**D**) IgM (1160–1028 cm^−1^), the outputs of both populations lump together; moreover, none demarcation line between the groups is observed, mixing the healthy and COVID-19 populations.

Figure 6 shows the score plots obtained employing the MLRM with the spectra of healthy and COVID-19 groups. This MLRM was done in four spectral regions: 1700–1600 cm^−1^ (amide I of proteins), and the regions related to IgG (1560–1464 cm^−1^) and IgM (1420–1289 cm^−1^, 1160–1028 cm^−1^). In the amide I region (Fig. 6A), the outputs data are very compact for each population, and the distance between them is well defined, allowing their discrimination, even though some data from the COVID-19 group are badly grouped located in the healthy group. In the same way, in the IgG region (Fig. 6B), the data are also grouped in two populations, allowing the discrimination between healthy and COVID-19 groups; nevertheless, some data of both populations are ungrouped locating COVID-19 data in the healthy group. In contrast, in the IgM regions (Fig. 6C,D), the outputs are clustered, but the distance between the groups is not well defined, not allowing the identification of groups. Moreover, in the region 1160–1028 cm^−1^ (Fig. 6D), the outputs of both populations lump together, mixing the healthy and COVID-19 populations. Interestingly, the best regions that allowed the discrimination between COVID-19 and healthy groups were amide I and IgG.

Even though the MLRM graphs showed that the best regions to discriminate both populations were amide I and IgG, we calculated the sensibility, specificity, and exactitude metrics, shown in Table 5.

**Table 5:** Sensibility, specify, and exactitude of the multiple linear regression model (MLRM).

Region	Interval (cm^−1^)	Sensibility (%)	Specificity (%)	Exactitude (%)
Amide I of proteins	1700–1600	99.2	100	99.6
IgG	1560–1464	99.2	100	99.6
IgM	1420–1289	99	99.9	99.5
	1160–1028	77.1	93.2	87

The results obtained in Table 5 indicate that the greatest affectations attributable to the SARS-CoV-2 are observable in the amide I and IgG regions since without considering the other regions of the FTIR spectrum and individually analyzing the vibrations of these subregions of the saliva spectra, it was possible to obtain the best metrics of sensitivity (99.2%), specificity (100%), and accuracy (99.6%) for both subregions.

Equally or even more important than the values presented in Table 5 is the analysis of the behavior of the outputs of the classification model (MLRM). Therefore, in Table 6, we provide a brief analysis of these values.

**Table 6:** MLRM output analysis.

Region	Interval (cm^−1^)	Healthy		COVID-19	
		Output range	SD^a^	Output range	SD^a^
Amide I of proteins	1700–1600	0.62–1.39	0.06	0.95–2.15	0.09
IgG	1560–1464	0.44–1.46	0.07	0.94–2.28	0.10
IgM	1420–1289	0.49–1.53	0.12	0.79–2.37	0.14
	1160–1028	0.25–1.86	0.21	0.81–3.63	0.32

^a^Standard deviation.

However, the detailed analysis of the output values obtained by the MLRM (Table 6) indicates that using this model built from the vibrations of the amide I region, better results with a larger population are shown, this due to that the standard deviation of the output values for both populations (COVID-19 and healthy groups) are lower compared to the standard deviations of the IgG region.

In this sense, we determined that it is possible to discriminate both populations by analyzing the region associated with amide I; nevertheless, it was also possible to misidentify six COVID-19 patients.

## Discussion

Considering the previous knowledge that virus infections provoke changes in the structures of biomolecules, in this research, we examined the FTIR spectra of COVID-19 and healthy patients, seeking the discrimination between these two populations through the analysis of FTIR spectra and an MLRM. Although ATR-FTIR is not used as a diagnosis technique, several authors have reported the use of FTIR for virus detection; for example, Erukhimovitch et al. in 2005, stated that it is possible to apply FTIR microscopy as a sensitive and effective assay for the detection of cells infected with various members of the herpes family of viruses and retroviruses^23^. Furthermore, Lee-Montiel et al., in 2011, evaluated the utility of FTIR spectroscopy for rapid detection of infective virus particles poliovirus in cell cultures^24^, and Santos et al. in 2020, reported several spectral features changes for hepatitis infected patients^17^. Additionally, nowadays, Banerjee et al. developed a predictive algorithm for COVID-19 disease stratification into severe and non-severe COVID-19 through ATR-FTIR spectra^25^. Therefore, in the search to propose new techniques that allow detecting the SARS-CoV-2 virus, FTIR spectroscopy has been considered in this research.

In the analysis of the COVID-19 population characteristics, although Peckham et al. have demonstrated that there is no difference in the proportion of males and females infected with SARS-CoV-2^26^, in this research, we documented that 160 (62.7%) men integrated the COVID-19 population, and 95 (37.3%) women, this probably due to the samples were obtained from hospitalized patients. Furthermore, the same authors declared that males face higher odds of intensive therapy unit (ITU) admission and death than females.

About the age, even though Hu et al. reported that it appears that all ages of the population are susceptible to SARS-CoV-2 infection, the median age of infection is around 50 years^27^, which was also observed in this research, once the average age was 54.3 ± 14.7 years.

As previously mentioned, concerning vital signs in the COVID-19 group, the only altered vital sign was the SaO_2_, showing a mean of 90%. Nevertheless, it is mandatory to remember that these patients were hospitalized, being one of the main criteria for hospitalization besides the evidence of pulmonary affection through computed tomography the low PO_2_, which entails a low SaO_2_. Furthermore, Hu et al. have reported that the most common symptoms in COVID-19 patients are fever, dry cough, and fatigue in patients less than 50 years, adding dyspnea in patients over 60 years^27^. Likewise, we found that this research's main reported symptoms were cough, dyspnea, headache, and fever.

About comorbidities, as previously mentioned, obesity, diabetes, and hypertension were the most reported entities in this study. Thus, these results agree with Ortiz-Brizuela et al., Berumen et al., and Petrova et al., who declared that the pathologies above are the main risk of COVID-19 infection and hospitalization^28–30^.

Regarding the blood group, even though Zhao et al. have reported that the blood group O is associated with a lower risk for the infection compared with non-O blood groups^31^, in this research, the main blood type was O, probability due to this blood type is the most common in Mexico^32^, country where this research took place.

Velavan and Meyer have declared about the laboratory blood tests that CRP, d-dimers, ferritin, cardiac troponin, and IL-6 could be used in risk stratification to predict severe and fatal COVID-19 in the hospitalized patient^33^. In this study, we observed that the values of neutrophiles, glucose, CRP, LDH, fibrinogen, d-dimer, and ferritin were increased, i.e., the patients that integrated this study presented three of the laboratory risks mentioned by Velavan, probably due to these patients were hospitalized because they required specialized medical attention. As expected, we detected neutrophilia, as it is known the primary function of the neutrophils is clearance of pathogens and debris through phagocytosis, the liberation of neutrophil extracellular traps is needed for viral infection inactivation and restriction of virus replication, been the neutrophils the first cell recruitment in COVID-19^34^. In addition, hypoxia and hypocapnia are seen in severe COVID-19 cases; Wang et al. reported a median PaO_2_ of 68 mmHg and a median of PaCO_2_ of 34 mmHg in 138 COVID-19 patients^35^, results that are similar to the ones obtained in this research (PaO_2_ 66 mmHg, and PaCO_2_ 31.1 mmHg).

On the other hand, regarding FTIR spectra, the obtained spectra were similar to those reported by Caetano et al., showing characteristics of biological samples^16^. However, the population evaluated by Caetano et al. was informed to abstain from food and caffeine products for at least two hours before the saliva collection and rinse out their mouths with distilled water. Contrary, in this study, a fasting period of at least 8 h was required, and an exclusion criterion was patients who had brushed or rinsed the oral cavity with mouthwash before sampling.

As previously mentioned, in the FTIR spectra analysis, a slight displacement, as well as a decrease in the absorbance in the regions of amide I and amide II, were exhibited in the COVID-19 group, which may be attributed to a decrease in protein production, which corresponds to that reported by Bojkova et al., who observed a decrease in the expression of proteins, especially those related to cholesterol metabolism in CaCo-2 cells infected by SARS-CoV-2^36^. In the same way, Bouhaddou et al. reported a decrease in the abundance of host proteins and a predominance of viral proteins, which is consistent with the mechanisms reported by other viruses in the inhibition of protein translation of the guest^37^. Similar to that found in Vero cells infected by herpes viruses, protein synthesis and cellular metabolism decrease in the initial stages of infection consuming cellular metabolites such as nucleotides, amino acids, and cellular enzymes^36–38^. Highlighting that Barauna et al. reported a decrease in the peak related to amide I in saliva combined with inactive SARS-CoV-2 virus compared to saliva without infection^39^.

In the same way, the peaks at 1240 cm^−1^ and 1076 cm^−1^, which are related to phosphorylated molecules, are increased in COVID-19 patients, respect to healthy patients. Bouhaddou et al. reported an increase in phosphorylated proteins with a decrease in protein abundance and hyperphosphorylation of the CK2 and p38 MAPK pathways related to cytokine production^37^, which is also consistent with that reported by Diamond et al.^40^. Moreover, Erukhimovitch et al. reported an increase in the peak at 1240 cm^−1^ in cells infected with the herpes virus^38^.

About the band at 1030 cm^−1^ attributed to carbohydrates (including glucose, fructose, and glycogen), it is known that the SARS-CoV-2 spike glycoprotein (S-protein) is occupied by 66 glycosylation sites, each of which can be occupied by up to 10 different glycans (carbohydrates) upon infection. After the attack of viruses in the human body through the respiratory tract, they usually utilize sugar chains (glycans) present on the surface of host cells. Thus, the virus is covered by glycans resistant to mutation through its development process^41^. In this research, the band correlated to carbohydrates showed a higher expression in the COVID-19 group, probably due to the high concentration of spike glycoprotein.

The region of nucleic acids (1100–850 cm^−1^) showed a higher expression in the COVID-19 group, probably because SARS-CoV-2 can be detected in more than 95% of saliva samples. Moreover, the virus can be cultured from saliva, which means that the virus is present in this biofluid. Besides, virus detection in saliva has also been used to monitor viral load dynamics over time^42^.

About the immune response, it has been declared that the combination of IgG and IgM achieves an overall sensitivity of 87.8% and specificity of 98.9% for detecting SARS-CoV-2; nevertheless, the complexity of the humoral response in COVID-19 is not fully elucidated, and the relevance of the SARS-CoV-2 antibody response for the long-term clinical outcome of viral clearance is still lacking. Furthermore, some authors have declared that the reported time to IgM positivity ranges from 5 to 10 days following disease onset, whereas IgG positivity occurs between 13 and 21 days. Moreover, others have stated that the earliest detection of IgM was at 5 days post symptom onset, and the earliest detection of IgG was at 7 days post symptom onset^43–45^.

In the same way, it has been reported that IgA plays an essential role in mucosal immunity, being the most crucial immunoglobulin to fight infectious pathogens in the respiratory system^46^. Furthermore, it has been stated that salivary testing is the most convenient way to measure IgA, the reason by which it has been used to characterize mucosal immune responses to many viral infections such as SARS, MERS, influenza, HIV, and RSV. Serum IgA has been detected in COVID-19 patients and appears to be detectable earlier than IgM or IgG antibodies, possibly as early as two days after onset of symptoms, suggesting that IgA may be the first antibody to appear in response to SARS-CoV-2 infection^47^. In this research, changes in absorbance in the areas related to IgG (1560–1464 cm^−1^), IgM (1420–1289 cm^−1^, 1160–1028 cm^−1^), and IgA (1285–1237 cm^−1^) were observed, noticing a higher absorbance in the spectra of COVID-19 group, which is concordant with all those mentioned above.

On the other hand, the second derivative spectra of COVID-19 patients in the amide I proteins region (1700–1600 cm^−1^) showed an absorbance decrease and a displacement, suggesting changes in the protein structures and less content of these secondary structures. According to Usoltsev et al., who studied the secondary structure changes in human serum albumin under various denaturation conditions reported that the ranges herein described are attributed to β-turns (1689–1660 cm^−1^), α-helices (1660–1650 cm^−1^), β-sheets (1639–1620 cm^−1^), and intermolecular β-sheets (1619–1610 cm^−1^) in the secondary structure of the human serum albumin^22^. This protein has been studied as a key in COVID-19 clinical evolution; Viana-Llamas et al. have declared that hypoalbuminemia is a predictor of mortality. Hypoalbuminemia is associated with AN inflammatory response in critical illness; this due to the cytokines and chemokines released induce an increase in capillary leakage, altering the distribution of albumin between intravascular and extravascular compartments. Viana-Llamas et al. reported that in COVID-19 patients, serum albumin concentration media at the moment of hospital admission was 34.4 ± 4.0 g/L, and in deceased patients 32.3 ± 4.1 g/L^48^, which is concordant to the results reported in this research once the second derivate analysis of the bands associated to albumin showed a decrement in the COVID-19 group. Moreover, the laboratory blood test reported hypoalbuminemia (3.3 g/dL) at the hospital admission moment, remembering that the saliva sample was collected in the first 3 weeks of hospitalization, that is, probably if the blood sample had been taken at the same time as the saliva, the albumin concentration could have been lower.

Furthermore, Diamond et al. declared a decrease in the expression of the mRNA of ACE2 and IL-6 in saliva samples, which would correspond to the decrease in the secondary structures reported by Meirson et al., who through a bioinformatic analysis described that the main secondary structure between the union of SARS-CoV-2 and ACE is the к-helix structure (polyproline II), followed by the α-helix and β-strand, changing the disulfide bonds^40,49^. Moreover, Giubertoni et al. assigned the peak at 1619 ± 2 cm^−1^ as helical conformation and 1659 ± 2 cm^−1^ as α-helix, which are also diminished herein^50^.

As expected, the immunoglobulins content showed that the COVID-19 group expressed a higher IgA, IgM, and IgG content than the healthy group. Moreover, when comparing the expression of these in the COVID-19 group, it can be observed that the IgA was the least immunoglobin expressed, followed by the IgM; being the IgG the most expressed immunoglobulin, which may be attributed to that most of the samples were collected at day 9.24 after PCR diagnosis, and according to the aforementioned the IgM is detected 5 days post symptom onset. On the other hand, the earliest detection of IgG is at 7 days post symptom onset. Nevertheless, some samples were obtained on the first day of symptoms so that, IgA was detected in this population.

When comparing DNA and nucleic acids content, the COVID-19 group showed a higher content of these molecules. Besides aforementioned about the presence of the virus in saliva, Paolini et al. have stated that SARS-CoV-2 promotes cell death^51^, and Zelig et al. have declared that in necrotic cell death, the DNA is completely unwound, the reason by which 100% of the DNA is visible to IR at this stage, observing an increase of ∼ 65% in DNA absorbance in necrosis compared to the control. Moreover, they also reported a decrease in the random coil structure of the total protein^52^, similarly to the COVID-19 group of this research. In addition, it also agrees to the results observed in the second derivative of proteins, where a decreased absorption at the range of random coil in the COVID-19 group is observed, as well as an increment of the bands related to nucleic acids. Moreover, Wood et al. reported that the band at 1078 cm^−1^ among others allowed to distinguish the positive from the negative COVID-19 samples^53^; this band was found at 1076 cm^−1^ in this research, showing a higher absorbance in the COVID-19 group.

On the other hand, as previously mentioned, the characterization of two or more populations from the analysis of the FTIR spectra of their individuals is not an easy task; in a more complex sample, it will be more complicated to find characteristic patterns of the population. This because the links of the different components could overlap with the characteristic component links of each sample. Moreover, the nature of the samples (fluid or tissues, cells, among others) has its particularities.

Different methodologies have been proposed to identify populations from the analysis of FTIR spectra, facilitating the adoption of a classification method by allowing experimentation to focus only on the most promising. In this sense, in another work, we first experimented with linear classification models to discriminate COVID-19 patients, although these models were affected by the overlap of the spectra due to the variances of the absorbances/transmittances of the populations; this problem can be overcome by having a large population thanks to the central limit theorem. In this work, we discriminated against our groups employing an MLRM, which was validated employing a LOOCV according to our previous research.

The absorbance variations and principally the peak displacement associated with viral infections shown in Fig. 2A,B contributed to the excellent performance of MLRM. As we note in (1), the slope performs an essential role in MLRM models because a displacement in any peak means that one population has reached its maximum absorbance level while the other continues growing, so its sign is the opposite. Thus, our results presented in Fig. 6 suggest that the best region to identify possible virus carriers is the amide I of proteins (1700–1600 cm^−1^) to compact the outputs between the predictions of the same populations and the separation to the other one.

Enthought the spectra analysis allowed us to detect the molecular components that characterize a positive patient to SARS-CoV-2, and the data analysis through MLRM let us discriminate these patients from healthy persons, more assays need to be done, one of them should consider the time elapsed from the symptoms to the diagnosis and categorize this population. Another one should consider the diagnosis corroboration through the serological test (IgA, IgM, and IgG), correlating these results with the FTIR spectra.

Herein, we are proposing a new diagnosis strategy that could be used as screening due to its low cost; once this technique does not require consumables, recognizing as gold-standard diagnosis the RT-PCR. Nevertheless, there are large discrepancies about RT-PCR effectiveness. For example, Hellewell et al. have declared that the probability of a positive PCR test is 77% by four days after infection, decreasing to 50% by ten days after infection, reaching 0% by 30 days after infection, being the day 1–3 when the probability of detecting increase^54^. On the other hand, Jarrom et al. estimated a sensitivity of 87.8%^55^. However, we reached a sensibility of 99.2% and specificity of 100% in the amide I region, even though more studies need to be done in a more significant population.

## Conclusions

The present research established specific salivary vibrational modes employing ATR-FTIR spectroscopy and characterizing the COVID-19 biological fingerprint. These specific spectra can be used to detect possible carriers of the virus or patients who have presented the disease and retain some immunological respect. In any case, it is necessary to analyze and continue investigating the spectra in their different regions to determine their meaning with greater precision.

In addition, these spectra have allowed us to identify a suitable region for COVID-19 detection. By performing the MLRM, the number of variables decreased considerably, which would help us think about viable techniques or devices for diagnosing diseases faster and cheaper.

## Data Availability

All the generated data and the analysis developed in this study are included in this article.

## References

[1] Carvalho LFDCES, Nogueira MS (2020). Optical techniques for fast screening—Towards prevention of the coronavirus COVID-19 outbreak. Photodiagnosis Photodyn. Ther..

[2] WHO Coronavirus Disease (COVID-19) Dashboard. World Health Organ 2021 (accessed May 17 2021). https://covid19.who.int.

[3] COVID-19 Treatment Guidelines Panel. Coronavirus Disease 2019 (COVID-19) Treatment Guidelines. National Institutes of Health (accessed May 17, 2021). https://www.covid19treatmentguidelines.nih.gov/. 34003615

[4] Afzal A (2020). Molecular diagnostic technologies for COVID-19: Limitations and challenges. J. Adv. Res..

[5] Chang FY (2020). Immunologic aspects of characteristics, diagnosis, and treatment of coronavirus disease 2019 (COVID-19). J. Biomed. Sci..

[6] Santaella-Tenorio J (2020). SARS-CoV-2 diagnostic testing alternatives for Latin America. Colomb. Med..

[7] Yang H, Yang S, Kong J, Dong A, Yu S (2015). Obtaining information about protein secondary structures in aqueous solution using Fourier transform IR spectroscopy. Nat. Protoc..

[8] Ferreira ICC (2020). Attenuated total reflection-fourier transform infrared (ATR-FTIR) spectroscopy analysis of saliva for breast cancer diagnosis. J. Oncol..

[9] Melián-Rivas A, Calcumil-Herrera P, Boin-Bakit C, Carrasco-Soto R (2020). Detección de COVID-19 (SARS-CoV-2) Mediante la Saliva: Una Alternativa Diagnóstica poco Invasiva. Int. J. Odontostomat..

[10] Farshidfar N, Hamedani S (2020). The potential role of smartphone-based microfluidic systems for rapid detection of COVID-19 using saliva specimen. Mol. Diagn. Ther..

[11] Zhang W (2020). Molecular and serological investigation of 2019-nCoV infected patients: Implication of multiple shedding routes. Emerg. Microbes Infect..

[12] To KK (2020). Temporal profiles of viral load in posterior oropharyngeal saliva samples and serum anti-body responses during infection by SARS-CoV-2: An observational cohort study. Lancet Infect. Dis..

[13] To KK (2020). Consistent detection of 2019 novel coronavirus in saliva. Clin. Infect. Dis..

[14] Wang A, Wang CP, Tu M, Wong DTW (2016). Oral biofluid biomarker research: Current status and emerging frontiers. Diagnostics.

[15] Wang W (2020). Detection of SARS-CoV-2 in different types of clinical specimens. JAMA.

[16] Caetano PC, Strixino JF, Raniero L (2015). Analysis of saliva by Fourier transform infrared spectroscopy for diagnosis of physiological stress in athletes. Res. Biomed. Eng..

[17] Santos MCD, Morais CLM, Lima KMG (2020). ATR-FTIR spectroscopy for virus identification: A powerful alternative. Biomed. Spectrosc. Imaging.

[18] Mata-Miranda MM (2019). Characterization of the biological fingerprint and identification of associated parameters in stress fractures by FTIR spectroscopy. BioMed. Res. Int..

[19] Benezzeddine-Boussaidi L, Cazorla G, Melin AM (2009). Validation for quantification of immunoglobulins by Fourier transform infrared spectrometry. Clin. Chem. Lab. Med..

[20] Gautam R (2016). Molecular profiling of sepsis in mice using fourier transform infrared microspectroscopy. J. Biophotonics.

[21] Raschka, S. Model Evaluation, Model Selection, and Algorithm Selection in Machine Learning. ArXiv::1811.12808v3 (2020).

[22] Usoltsev D, Sitnikova V, Kajava A, Uspenskaya M (2019). Systematic FTIR spectroscopy study of the secondary structure changes in human serum albumin under various denaturation conditions. Biomolecules.

[23] Erukhimovitch V, Talyshinsky M, Souprun Y, Huleihel M (2005). FTIR microscopy detection of cells infected with viruses. Methods Mol. Biol..

[24] Lee-Montiel FT, Reynolds KA, Riley MR (2011). Detection and quantification of poliovirus infection using FTIR spectroscopy and cell culture. J. Biol. Eng..

[25] Banerjee A (2021). Rapid classification of COVID-19 severity by ATR-FTIR spectroscopy of plasma samples. Anal. Chem..

[26] Peckham H (2020). Male sex identified by global COVID-19 meta-analysis as a risk factor for death and ITU admission. Nat. Commun..

[27] Hu B, Guo H, Zhou P, Shi ZL (2021). Characteristics of SARS-CoV-2 and COVID-19. Nat. Rev. Microbiol..

[28] Ortiz-Brizuela E (2020). Clinical and epidemiological characteristics of patients diagnosed with COVID-19 in a Tertiary Care Center in Mexico City: A prospective cohort study. Rev. Investig. Clin..

[29] Berumen J (2020). Risk of infection and hospitalization by Covid-19 in Mexico: A case-control study. medRxiv..

[30] Petrova D (2020). Obesity as a risk factor in COVID-19: Possible mechanisms and implications. Aten. Primaria.

[31] Zhao J (2020). Relationship between the ABO Blood Group and the COVID-19 Susceptibility. medRxiv..

[32] Canizalez-Román A (2018). Blood groups distribution and gene diversity of the ABO and Rh (D) loci in the Mexican population. Biomed Res. Int..

[33] Velavan TP, Meyer CG (2020). Mild versus severe COVID-19: Laboratory markers. Int. J. Infect. Dis..

[34] Borges L, Pithon-Curi TC, Curi R, Hatanaka E (2020). COVID-19 and neutrophils: The relationship between hyperinflammation and neutrophil extracellular traps. Mediat. Inflamm..

[35] Wang D (2020). Clinical characteristics of 138 hospitalized patients with 2019 novel coronavirus-infected pneumonia in Wuhan, China. JAMA.

[36] Bojkova D (2020). Proteomics of SARS-CoV-2-infected host cells reveals therapy targets. Nature.

[37] Bouhaddou M (2020). The global phosphorylation landscape of SARS-CoV-2 infection. Cell.

[38] Erukhimovitch V, Bogomoln E, Huleihil M, Huleihel M (2011). Infrared spectral changes identified during different stages of herpes viruses infection in vitro. Analyst.

[39] Barauna VG (2021). Ultrarapid on-site detection of SARS-CoV-2 infection using simple ATR-FTIR spectroscopy and an analysis algorithm: High sensitivity and specificity. Anal. Chem..

[40] Diamond G (2020). Examination of gene expression in saliva samples from COVID-19 patients to study the host defense response against SARS-CoV-2 in the oral cavity. Mol. Oral Microbiol..

[41] Kumbhar PS, Pandya AK, Manjappa AS, Disouza JI, Patravale VB (2021). Carbohydrates-based diagnosis, prophylaxis and treatment of infectious diseases: Special emphasis on COVID-19. Carbohydr. Polym. Technol. Appl..

[42] Herrera LA (2021). Saliva is a reliable and accessible source for the detection of SARS-CoV-2. Int. J. Infect. Dis..

[43] Suhandynata RT (2020). Longitudinal monitoring of SARS-CoV-2 IgM and IgG seropositivity to detect COVID-19. J. Appl. Lab. Med..

[44] Secchi M (2020). COVID-19 survival associates with the immunoglobulin response to the SARS-CoV-2 spike receptor binding domain. J. Clin. Investig..

[45] de Assis RR (2021). Analysis of SARS-CoV-2 antibodies in COVID-19 convalescent blood using a coronavirus antigen microarray. Nat. Commun..

[46] Chao YX, Rötzschke O, Tan EK (2020). The role of IgA in COVID-19. Brain Behav. Immun..

[47] Varadhachary A (2020). Salivary anti-SARS-CoV-2 IgA as an accessible biomarker of mucosal immunity against COVID-19. medRxiv..

[48] Viana-Llamas MC (2021). Hypoalbuminemia on admission in COVID-19 infection: An early predictor of mortality and adverse events. A retrospective observational study. Med. Clin..

[49] Meirson T, Bomze D, Markel G (2021). Structural basis of SARS-CoV-2 spike protein induced by ACE2. Bioinformatics.

[50] Giubertoni G, Meister K, DeVries AL, Bakker HJ (2019). Determination of the solution structure of antifreeze glycoproteins using two-dimensional infrared spectroscopy. J. Phys. Chem. Lett..

[51] Paolini A (2021). Cell death in coronavirus infections: Uncovering its role during COVID-19. Cells.

[52] Zelig U, Kapelushnik J, Moreh R, Mordechai S, Nathan I (2009). Diagnosis of cell death by means of infrared spectroscopy. Biophys. J..

[53] Wood BR (2021). Infrared based saliva screening test for COVID-19. Angew. Chem. Int. Ed. Engl..

[54] Hellewell J (2021). Estimating the effectiveness of routine asymptomatic PCR testing at different frequencies for the detection of SARS-CoV-2 infections. BMC Med..

[55] Jarrom D (2020). Effectiveness of tests to detect the presence of SARS-CoV-2 virus, and antibodies to SARS-CoV-2, to inform COVID-19 diagnosis: A rapid systematic review. BMJ Evid. Based Med..

